# Serum sLYVE-1 is not associated with coronary disease but with renal dysfunction: a retrospective study

**DOI:** 10.1038/s41598-019-47367-2

**Published:** 2019-07-25

**Authors:** Daopeng Dai, Chunkai Huang, Jinwei Ni, Zhenbin Zhu, Hui Han, Jinzhou Zhu, Ruiyan Zhang

**Affiliations:** Department of Cardiology, Rui Jin Hospital, Shanghai Jiaotong University School of Medicine, Shanghai, People’s Republic of China

**Keywords:** Chronic kidney disease, Predictive markers, Cardiovascular diseases

## Abstract

Recent evidence has indicated that the lymphatic vessel endothelial hyaluronan receptor (LYVE-1) is implicated in chronic inflammation and the lymphatic immune response. The soluble form of LYVE-1 (sLYVE-1) is produced by ectodomain shedding of LYVE-1 under pathological conditions including cancer and chronic inflammation. In this study, 1014 consecutive patients who underwent coronary angiography from May 2015 to September 2015 were included to investigate whether serum sLYVE-1 is associated with coronary artery disease (CAD) and its concomitant diseases includes chronic kidney disease (CKD). Results showed that there was no significant difference in sLYVE-1 levels between patients with CAD and without. However, a significantly higher level of sLYVE-1 was seen in patients with renal dysfunction compared to those with a normal eGFR. Results were validated in a separate cohort of 259 patients who were divided into four groups based on their kidney function assessed by estimated glomerular filtration rate (eGFR). **S**imple bivariate correlation analysis revealed that Lg[sLYVE-1] was negatively correlated with eGFR (r = −0.358, *p* < 0.001) and cystatin C (r = 0.303, *p* < 0.001). Multivariable logistic regression analysis revealed that the increase in Lg[sLYVE-1] was an independent determinant of renal dysfunction (odds ratio = 1.633, *p* = 0.007). Therefore, renal function should be considered when serum sLYVE-1 is used as a biomarker for the detection of pathological conditions such as chronic inflammation and cancer. Further study is required to elucidate the exact role of sLYVE-1 in renal function.

## Introduction

The lymphatic system, known as the second circulatory system, is indispensable in draining interstitial fluid from tissues and returning it to blood circulation^1^. Studies have shown that acute inflammatory reactions and chronic inflammatory diseases are accompanied by both the expansion of preexisting lymphatic vessels (lymphatic hyperplasia) and the growth of new lymphatic vessels (lymphangiogenesis), indicating a role for the lymphatic system in immune surveillance and the inflammatory process, including that which arises in the context of coronary artery disease (CAD)^2–6^.

LYVE-1, a major receptor for hyaluronic acid (HA) on the lymph vessel wall, has been widely used as a uniquely powerful marker for lymph vessels^7^. During the last decade, accumulating evidence has provided insight into the role of LYVE-1 in lymphangiogenesis and immune responses. LYVE-1 has been reported as a mediator of lymphangiogenesis induced by low-molecular weight HA^8^. Additionally, it has been shown that, in cultured primary lymphatic endothelial cells, surface expression of LYVE-1 is rapidly and reversibly lost after exposure to tumor necrosis factor-α (TNF-α) and tumor necrosis factor-β (TNF-β), occurring via internalization and lysosomic degradation of LYVE-1 coupled with a shutdown in gene expression^9^.

Recently, ectodomain shedding of LYVE-1 has been found to occur in lymphatic vessels during chronic inflammation^10^. A potential role for soluble LYVE-1 (sLYVE-1), which contains the intact binding domain for its ligands, has been reported in the inhibition of LYVE-1 signaling^11^. Although the exact function of sLYVE-1 in normal physiology and pathological conditions is not yet clear, the association between sLYVE-1 and several diseases, including rheumatoid arthritis and cancer, has been explored^12,13^.

Given the important role of the lymphatic system in reversing cholesterol transport during the development of atherosclerosis, an association between serum sLYVE-1 levels and CAD severity was hypothesized^14,15^. In this study, we aimed to investigate whether serum sLYVE-1 is related to CAD as well as its concomitant diseases including hypertension, dyslipidemia, diabetes, and chronic renal dysfunction, all of which are known to be conditions associated with chronic inflammation^16–20^.

## Methods

The study protocols for cohort 1 and cohort 2 were approved by the Ethics Committee of Rui Jin Hospital, Shanghai Jiaotong University School of Medicine. Written informed consent was obtained from all participants. All experiments and methods were performed in accordance with relevant guidelines and regulations.

### Study population

1014 consecutive patients subjected to coronary angiography (CAG) from May 2015 to September 2015 were included in this study (referred to as cohort 1 below). To avoid confounding data, patients with acute myocardial infarction, acute and chronic viral or bacterial infection, tumors, type 1 diabetes mellitus, or rheumatoid arthritis and other connective tissue diseases were excluded. Results were validated in a separate cohort of 259 patients with or without renal disfunction who subjected to coronary angiography for the first time from February 2017 to November 2017 (referred to as cohort 2 below). Patients with acute myocardial infarction, acute and chronic viral or bacterial infection, tumors, type 1 diabetes mellitus, connective tissue diseases, radiographic contrast nephropathy and hemodynamic instability were excluded. Patients in cohort 2 were divided into four groups according to estimated glomerular filtration rate (eGFR) (eGFR < 30 mL/min/1.73 m^2^, n = 37; 30 mL/min/1.73 m^2^ ≤ eGFR < 60 mL/min/1.73 m^2^, n = 74;60 mL/min/1.73 m^2^ ≤ eGFR < 90 mL/min/1.73 m^2^, n = 74; 90 mL/min/1.73 m^2^ ≤ eGFR, n = 74). Data recorded for each patient included a detailed medical and family history, results of routine laboratory tests, and details regarding concomitant diseases.

### Definitions

Type 2 diabetes mellitus (T2DM) was identified if two fasting plasma glucose levels were ≥7.0 mmol/L, if two 2 h postprandial plasma glucose readings were ≥11.1 mmol/L after a glucose load of 75 g, if two casual glucose readings were ≥11.1 mmol/L, or if the patient was taking oral hypoglycemic drugs or parenteral insulin^21^. CAD patients were documented based on angiographic examination (luminal diameter narrowing ≥50%)^22^. Patients with significant CAD were further classified according to the number of diseased coronary arteries (1-, 2-, or 3-vessel disease). eGFR was calculated by using a modified CKD-EPI equation as follows: eGFR_CKD-KPI_ = 141*0.993^age*[serum creatinine/79.56]^(–0.411)(−1.209 if serum creatinine ≥79.56μ mol/L) for men, and eGFR_CKD-KPI_ = 141*0.993^age*[serum creatinine/61.88]^(–0.329)(−1.209 if serum creatinine ≥61.88 μmol/L) for women. Preserved and impaired renal function were defined as eGFR_CKD-KPI_ ≥ 60 mL/min/1.73 m^2^ and eGFR_CKD-KPI_ < 60 mL/min/1.73 m^2^, respectively.

### Coronary angiography and gensini score

Selective coronary angiography was performed via the femoral or radial artery by interventional cardiologists blinded to the study protocol. Significant CAD was diagnosed visually if narrowing of the luminal diameter ≥50% was present in a major epicardial coronary artery, and left main coronary artery narrowing ≥50% was considered 2-vessel disease^22^. Gensini score was calculated according to the published method^23^.

### Biochemical investigation

Blood samples were collected after overnight fasting from all patients. Serum cystatin-c, as well as creatinine, blood urea nitrogen, uric acid, total cholesterol, Triglyceride, low-density lipoprotein-cholesterol, high-density lipoprotein cholesterol and hsCRP were measured with standard laboratory techniques in our hospital laboratory. Serum sLYVE-1 level was determined with a commercially available ELISA kit (Human LYVE-1 DuoSet ELISA, DY2089, R&D System, Minneapolis, MN) according to the instructions. Once collected, the samples were immediately immersed in ice and centrifuged within 30 minutes at 12,000 rpm for 10 min to obtain platelet-poor serum. All samples were stored at −80 °C before analysis.

### Statistical analysis

Continuous variables were presented as mean ± standard deviation (SD), while discrete data were presented as median with a 25–75% range. Categorical data were summarized as frequency with percentage. For categorical clinical variables, we evaluated differences between groups with the chi-square test followed by Bonferroni’s correction to account for multiple comparisons. For continuous variables, we evaluated the presence of a normal distribution with the Kolmolgorov–Smirnov test, and applied logarithmic transformations on continuous variables displaying a non-normal distribution of sLYVE-1 levels. Continuous variables were compared using independent t-test between two groups. And we analyzed differences among patient groups by one-way analysis of variance (ANOVA) or the Kruskal–Wallis analysis, followed by post-hoc analysis. The correlation between variables was determined by the Pearson correlation tests or Spearman correlation tests as appropriate. In multivariable stepwise logistic regression analysis, conventional risk factors and Lg[sLYVE-1] were adjusted for the assessment of renal dysfunction. A 2-tailed *p* < 0.05 was considered statistically significant. Statistical analysis was performed with SPSS 22.0 for Windows (SPSS, Inc., Chicago, IL, USA). G*Power 3.1^24^ was used to conduct post hoc power analysis.

## Results

### Clinical characteristics and biochemical measurements

The baseline clinical characteristics and biochemical measurements of 1014 patients are presented in Table 1. Patients were further trisected into 3 groups according to serum sLYVE-1 levels (Table 2). As shown, patients with high sLYVE-1 levels were much older, and the prevalence of diabetes mellitus and chronic kidney disease (CKD) were significantly higher in this group. Fasting glucose, glycosylated hemoglobin, and creatinine concentration consistently increased in patients with higher sLYVE-1 levels, while eGFR decreased. Notably, the prevalence of hypertension and dyslipidemia showed no significant difference among the three groups.

**Table 1:** Baseline clinical characteristics and biochemical measurements of cohort 1.

cohort 1 (n = 1014)	
Male (%)	707 (69.7)
Age (y)	63.7 ± 10.1
BMI (kg/m^2^)	24.96 ± 3.52
Alcohol users (%)	113 (11.1)
Cigarette smoking (%)	349 (34.4)
Systolic (mmHg)	133.6 ± 20.4
Diastolic (mmHg)	75.2 ± 12.1
Diabetes mellitus	180 (17.8)
Hypertension (%)	644 (63.5)
Dyslipidemia (%)	129 (12.7)
CKD (%)	34 (3.4)
WBC (10^9^/L)	6.56 ± 2.08
Neutrophil%	58.88 ± 12.11
Lymphocyte%	27.85 ± 9.53
Fasting glucose (mmol/L)	5.40 ± 1.55
HbA1c (%)	6.16 ± 1.02
Total cholesterol (mmol/L)	3.92 ± 1.19
Triglyceride (mmol/L)	1.55 ± 0.97
HDL-C (mmol/L)	1.07 ± 0.29
LDL-C (mmol/L)	2.27 ± 0.97
BUN (mmol/L)	6.74 ± 17.78
Creatinine (umol/L)	84.39 ± 37.26
Uric acid (umol/L)	343.92 ± 96.54
NT-proBNP (pg/mL)	672.0 ± 2150.0
cTNI (ng/mL)	2.53 ± 28.39
eGFR (mL/min/1.73^2^)	80.64 ± 18.62
HsCRP (mg/L)	6.39 ± 24.89
AMI	117 (11.5)
**CAD**	
1-vessle disease	262 (25.8)
2-vessle disease	219 (21.6)
3-vessle disease	298 (29.4)
sLYVE-1 (ng/mL)	452.37 ± 212.13
Lg[sLYVE-1]	2.61 ± 0.20

Data are shown as number (%), mean ± SD. Abbreviations: AMI, acute myocardial infarction; BMI, body mass index; BUN, blood urea nitrogen; CAD, coronary artery disease; hsCRP, high sensitivity C reactive protein; HDL-C, high-density lipoprotein cholesterol; LDL-C, low-density lipoprotein cholesterol; sLYVE-1, soluble lymphatic vessel endothelial hyaluronan receptor-1; WBC, white blood cells.

**Table 2:** Baseline clinical characteristics and biochemical measurements of cohort 1 patients sorted into sLYVE-1 tertiles.

	lower	middle	higher	*P* value
	(sLYVE-1 < 344.05)	(344.05–498.11)	(LYVE-1 > 498.11)	
	(n = 338)	(n = 338)	(n = 338)	
Male (%)	243 (71.9)	238 (70.4)	226 (66.9)	0.343
Age (y)	62.2 ± 10.2	63.5 ± 10.3	65.4 ± 9.6	0.000
BMI (kg/m^2^)	25.10 ± 3.40	24.77 ± 3.22	25.00 ± 3.93	0.459
Alcohol users (%)	41 (12.1)	35 (10.4)	37 (10.9)	0.757
Cigarette smoking (%)	118 (34.9)	115 (34.0)	116 (34.3)	0.970
Systolic (mmHg)	132.7 ± 19.8	133.0 ± 20.1	135.1 ± 21.4	0.263
Diastolic (mmHg)	75.9 ± 12.0	74.6 ± 12.0	74.9 ± 12.3	0.320
Diabetes mellitus (%)	59 (17.5)	47 (13.9)	74 (21.9)	0.025
Hypertension (%)	215 (63.6)	207 (61.2)	222 (65.7)	0.487
Dyslipidemia (%)	42 (12.4)	38 (11.2)	49 (14.5)	0.438
CKD (%)	7 (2.1)	5 (1.5)	22 (6.5)	0.000
WBC (10^9^/L)	6.49 ± 2.15	6.53 ± 1.94	6.67 ± 2.14	0.473
Neutrophil%	58.09 ± 11.44	59.19 ± 12.51	59.34 ± 12.35	0.346
Lymphocyte%	28.82 ± 9.33	27.45 ± 9.49	27.28 ± 9.74	0.071
Fasting glucose (mmol/L)	5.33 ± 1.53	5.25 ± 1.14	5.61 ± 1.89	0.006
HbA1c%	6.13 ± 1.00	6.04 ± 0.82	6.30 ± 1.19	0.005
Total cholesterol (mmol/L)	3.89 ± 1.45	3.90 ± 1.04	3.96 ± 1.03	0.735
Triglyceride (mmol/L)	1.52 ± 0.94	1.55 ± 0.95	1.59 ± 1.02	0.663
HDL-C (mmol/L)	1.05 ± 0.27	1.07 ± 0.27	1.07 ± 0.31	0.538
LDL-C (mmol/L)	2.25 ± 1.14	2.26 ± 0.90	2.30 ± 0.84	0.782
BUN (mmol/L)	5.80 ± 1.95	8.26 ± 30.69	6.16 ± 2.33	0.153
Creatinine (umol/L)	82.29 ± 48.22	82.15 ± 19.80	88.70 ± 37.66	0.033
Uric acid (umol/L)	333.45 ± 87.52	342.57 ± 101.36	355.73 ± 99.19	0.010
cTNI (ng/mL)	4.56 ± 47.01	0.90 ± 6.45	2.00 ± 9.29	0.248
eGFR (mL/min/1.73 m^2^)	83.86 ± 17.25	81.70 ± 18.34	76.37 ± 19.47	0.000
hsCRP (mg/L)	5.81 ± 32.10	5.05 ± 15.74	8.39 ± 24.38	0.246
AMI (%)	42 (12.4)	29 (8.6)	46 (13.6)	0.101
CAD (%)	277 (74.0)	247 (61.3)	225 (64.7)	0.088
1-vessle disease (%)	93 (27.5)	78 (23.1)	91 (26.9)	
2-vessle disease (%)	53 (24.6)	74 (21.9)	62 (18.3)	
3-vessle disease (%)	101 (29.9)	95 (28.1)	102 (30.2)	
sLYVE-1 (ng/mL)	256.08 ± 61.69	413.17 ± 43.27	687.86 ± 183.79	0.000
Lg[sLYVE-1]	2.39 ± 0.13	2.61 ± 0.05	2.83 ± 0.10	0.000

Data are shown as number (%), mean ± SD. Abbreviations: AMI, acute myocardial infarction; BMI, body mass index; BUN, blood urea nitrogen; CAD, coronary artery disease; hsCRP, high sensitivity C reactive protein; HDL-C, high-density lipoprotein cholesterol; LDL-C, low-density lipoprotein cholesterol; sLYVE-1, soluble lymphatic vessel endothelial hyaluronan receptor-1; WBC, white blood cells.

### sLYVE-1 was not associated with CAD

**Figure 1:**
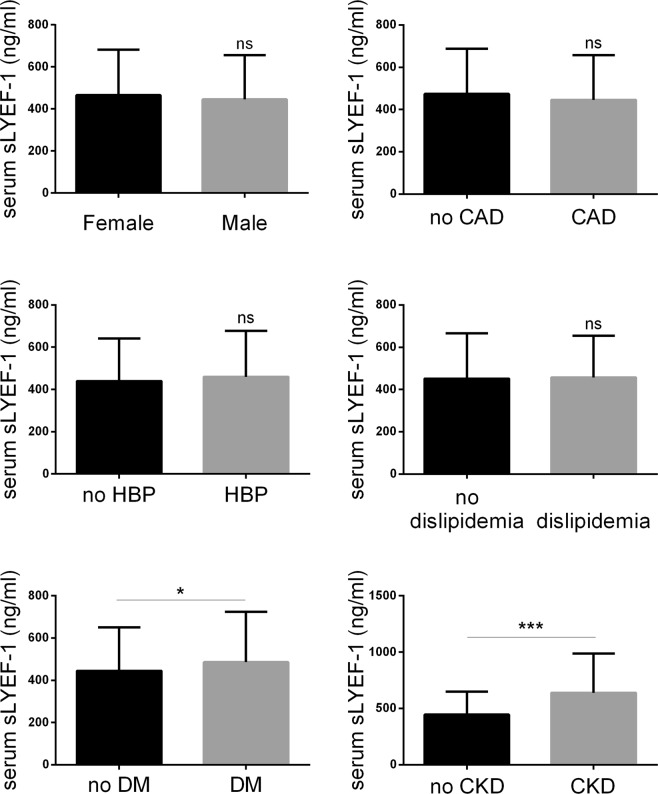
Serum sLYVE-1 levels in subgroups of cohort 1 patients. Comparison of sLYVE-1 levels were made between males and females (test power 0.282), and between subjects with hypertension and without (test power 0.297), with diabetes mellitus and without (test power 0.624), with dyslipidemia and without (test power 0.061), with CAD or without (test power 0.424), with CKD and without (test power 0.974). Results are presented as mean ± SD. Independent t-test was performed. *p < 0.05, ***p < 0.001. CAD, coronary artery disease; CKD, chronic kidney disease; DM, diabetes mellitus; HBP, high blood pressure.

**Figure 2:**
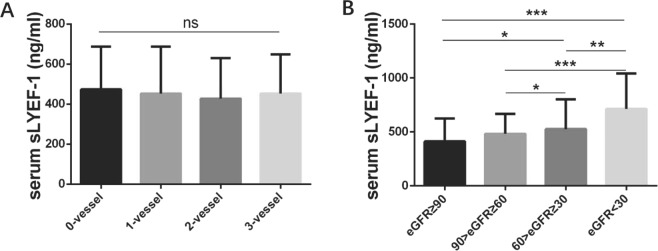
Serum sLYVE-1 levels in relation to the number of diseased coronary arteries and eGFR. Results are presented as mean ± SD. One-way ANOVA was performed followed by Turkey’s multiple comparisons tests. (**A**) According to angiography, patients in cohort 1 were divided into 4 groups: 0-vessel disease (n = 235), 1-vessel disease (n = 262), 2-vessel disease (n = 219), and 3-vessel disease (n = 298). Serum sLYVE-1 concentrations were not significantly different among groups. (**B**) Serum sLYVE-1 based on eGFR levels in cohort 2 patients. The serum level of sLYVE-1 was negatively associated with eGFR. *p < 0.05, **p < 0.01, ***p < 0.001. ns = not significant.

Serum sLYVE-1 concentrations showed no significant differences between subjects with hypertension and those without, with dyslipidemia and without, or between female and male subjects. Notably, the sLYVE-1 concentration was higher in subjects with diabetes mellitus. Contrary to our expectations there was no significant difference between patients with CAD and those without (Fig. 1). Patients with CAD were further classified according to the number of diseased coronary arteries. No significant differences of serum sLYVE-1 levels were found among four groups (*p* = 0.147 for ANOVA) (Fig. 2A). Partial correlation analysis showed that serum sLYVE-1 concentration was not significantly related to the number of diseased coronary arteries (Partial correlation coefficient = −0.044, p = 0.216, gender, age, BMI, SBP, Fast glucose, HbA1c, LVEF, hsCRP, HBP and DM were listed as covariables). However, a significantly higher sLYVE-1 level was seen in patients with CKD compared to those without (639.9 ± 348.2 vs. 445.9 ± 203.0, *p* = 0.003) (Fig. 1).

### sLYVE-1 was associated with renal dysfunction

An additional 259 patients were involved to further validate the association between sLYVE-1 level and renal function. Subjects were divided into four groups according to eGFR (Table 3). Significant differences with respect to gender existed amongst the four groups. The prevalence of hypertension and diabetes mellitus was higher in patients with lower eGFR, as was higher systolic blood pressure and hsCRP level. Notably, serum sLYVE-1 concentration increased as eGFR decreased (Table 3, Fig. 2B).

**Table 3:** Baseline clinical characteristics and biochemical measurements of cohort 2 patients sorted into eGFR quartiles.

	eGFR ≥ 90	90 > eGFR ≥ 60	60 > eGFR ≥ 30	30 > eGFR	*P* value
	(n = 74)	(n = 74)	(n = 74)	(n = 37)	
Male (%)	57 (77)	53 (71.6)	59 (79.7)	20 (54.1)	0.028
Age (y)	63.6 (13.5)	66.3 (12.5)	68.1 (11.6)	66.5 (13.7)	0.192
BMI (kg/m^2^)	24.41 (3.10)	25.48 (3.99)	24.09 (3.01)	24.60 (3.51)	0.080
Alcohol users (%)	4 (5.4)	5 (6.8)	6 (8.1)	3 (8.1)	0.917
Cigarette smoking (%)	20 (27.0)	17 (23.0)	17 (23.0)	10 (27.0)	0.905
Systolic (mmHg)	132.0 (16.7)	134.8 (19.7)	135.4 (23.3)	147.9 (32.9)	0.005
Diastolic (mmHg)	73.4 (11.8)	75.6 (11.2)	74.4 (13.6)	75.2 (12.7)	0.722
Hypertension (%)	36 (48.6)	54 (73.0)	53 (71.6)	34 (91.9)	0.000
Diabetes mellitus (%)	11 (14.9)	8 (10.8)	24 (32.4)	12 (32.4)	0.002
CAD (%)	52 (70.3)	47 (63.5)	56 (75.7)	26 (70.3)	0.455
WBC (10^9^/L)	6.22 (1.70)	6.15 (1.57)	7.09 (3.13)	7.24 (2.46)	0.011
Neutrophil%	59.11 (10.58)	59.91 (9.58)	63.99 (12.92)	65.77 (14.00)	0.006
Lymphocyte%	28.43 (9.02)	28.12 (8.80)	23.92 (9.34)	19.41 (8.71)	0.000
Fasting glucose (mmol/L)	5.45 (1.28)	5.15 (0.93)	6.65 (2.88)	5.87 (2.61)	0.000
HbA1c%	6.09 (0.93)	5.96 (0.88)	6.62 (1.37)	6.20 (0.90)	0.002
Total cholesterol (mmol/L)	4.10 (1.10)	3.97 (0.93)	3.87 (1.12)	4.06 (1.25)	0.610
Triglyceride (mmol/L)	1.44 (0.97)	1.28 (0.75)	1.77 (1.33)	1.47 (0.63)	0.033
HDL-C (mmol/L)	1.19 (0.32)	1.18 (0.24)	1.02 (0.26)	1.09 (0.30)	0.001
LDL-C (mmol/L)	2.29 (0.88)	2.29 (0.77)	2.24 (0.86)	2.41 (1.04)	0.825
BUN (mmol/L)	5.23 (1.36)	6.29 (1.86)	9.72 (4.34)	17.64 (6.75)	0.000
Creatinine (umol/L)	67.48 (9.77)	84.78 (12.30)	130.24 (32.43)	502.35 (338.39)	0.000
Uric acid (umol/L)	315.64 (76.05)	363.81 (75.90)	441.74 (148.09)	424.30 (120.57)	0.000
hsCRP (mg/L)	4.51 (16.23)	2.20 (3.47)	11.96 (35.14)	12.88 (27.17)	0.036
cystatin C (mg/L)	0.97 (0.16)	1.11 (0.24)	1.68 (0.46)	4.73 (2.12)	0.000
eGFR (mL/min/1.73 m^2^)	103.78 (11.60)	77.82 (8.65)	48.53 (8.96)	14.32 (9.02)	0.000
sLYVE-1 (ng/mL)	411.35 (214.11)	483.74 (183.96)	526.43 (275.95)	714.38 (327.53)	0.000
Lg[sLYVE-1]	2.56 (0.22)	2.65 (0.17)	2.67 (0.21)	2.81 (0.22)	0.000

Data are shown as number (%), mean ± SD. Abbreviations: BMI, body mass index; BUN, blood urea nitrogen; CAD, coronary artery disease; hsCRP, high sensitivity C reactive protein; HDL-C, high-density lipoprotein cholesterol; LDL-C, low-density lipoprotein cholesterol; sLYVE-1, soluble lymphatic vessel endothelial hyaluronan receptor-1; WBC, white blood cells.

### sLYVE-1 was an independent risk factor for renal dysfunction

To explore the correlation between sLYVE-1 and renal dysfunction, we analyzed sLYVE-1 as a log-transformed standardized continuous variable, Lg[sLYVE-1]. Simple bivariate correlation analysis revealed that Lg[sLYVE-1] negatively correlated with eGFR (r = −0.358, *p* < 0.001) and positively correlated with cystatin C (r = 0.303, *p* < 0.001). Moreover, an independent correlation between Lg[sLYVE-1] and eGFR (r = −0.274, *p* < 0.001) or cystatin C (r = 0.249, *p* < 0.001) was demonstrated in partial correlation analysis when adjusted for gender, age, BMI, SBP, fasting glucose, HbA1c, LVEF, hsCRP, HBP and DM (Table 4).

**Table 4:** Bivariate and partial correlation models for eGFR and cystatin C in cohort 2 patients.

Bivariate and Partial Correlation Models for eGFR and cystatin C in patients.									
eGFR	Bivariate Correlate		Partial Correlate		Cystatin C	Bivariate Correlate		Partial Correlate	
	r	*P* value	r	*P* value		r	*P* value	r	*P* value
Lg[sLYVE-1] per SD	−0.358	0.000	−0.274	0.000	Lg[sLYVE-1] per SD	0.303	0.000	0.249	0.001
gender	0.050	0.489	—	—	gender	0.008	0.914	—	—
Age	−0.216	0.002	—	—	Age	0.073	0.328	—	—
BMI	−0.060	0.407	—	—	BMI	−0.094	0.208	—	—
SBP	−0.242	0.001	—	—	SBP	0.150	0.043	—	—
Fasting glucose	−0.191	0.007	—	—	Fasting glucose	0.031	0.674	—	—
HbA1c	−0.139	0.053	—	—	HbA1c	0.023	0.755	—	—
hsCRP	−0.169	0.018	—	—	hsCRP	0.117	0.114	—	—
HBP	−0.287	0.000	—	—	HBP	0.185	0.013	—	—
DM	−0.230	0.001	—	—	DM	0.195	0.008	—	—

sLYVE-1 was analyzed as a log transformed continuous variable. The unit of change was specified as per SD ( = 0.215) for Lg (sLYVE-1). Gender, age, BMI, SBP, Fasting glucose, HbA1c, hsCRP, HBP and DM were involved as control factors in Partial Correlation Model. BMI, body mass index; SBP, systolic blood pressure; hsCRP, high sensitivity C reactive protein; HBP, high blood pressure; DM, diabetes mellitus.

**Figure 3:**
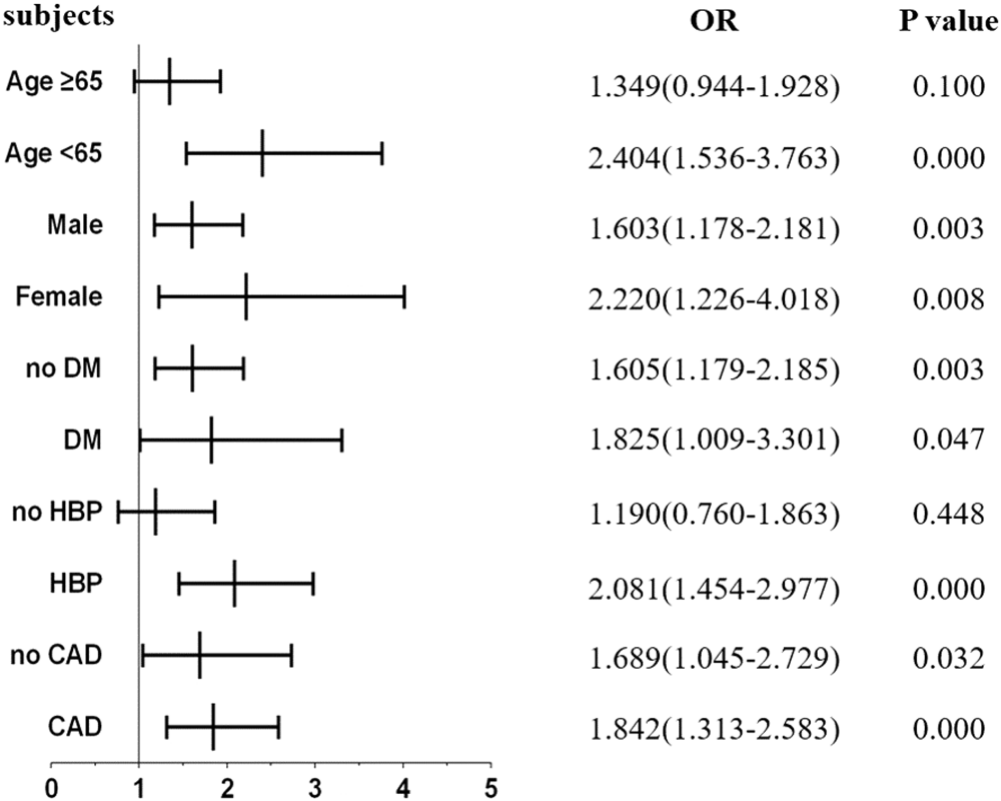
Forrest plots for renal dysfunction. Forrest plots (unadjusted) to analyze the predictive value of sLYVE-1 for eGFR < 60 mL/min/1.73 m^2^ in different subgroups of patients in cohort 2. sLYVE-1 is entered as a log-transformed continuous variable. DM, diabetes mellitus; HBP, high blood pressure; CAD, coronary artery disease.

To further establish the correlation between sLYVE-1 and renal dysfunction, patients were classified into two groups based on eGFR (eGFR < 60 mL/min/1.73 m^2^ and eGFR > 60 mL/min/1.73 m^2^). Univariate and multiple logistic regression analysis confirmed that Lg[sLYVE-1] was an independent predictor for the presence of renal dysfunction, both adjusted for age and gender and adjusted for the full model including age, gender, BMI, SBP, fasting glucose, HbA1c, LVEF, hsCRP, HBP and DM (Table 5). However, subgroup analysis by using a logistic regression model revealed Lg[sLYVE-1] to be a better predictor for diminished renal function in younger patients or those with HBP (Fig. 3).

**Table 5:** Univariate and multivariable linear regression models for renal dysfunction in cohort 2 patients.

Univariate and multivariable linear regression models for renal dysfunction						
	Unadjusted OR	*P* value	Adjusted for Model 1 OR	*P* value	Adjusted for Model 2 OR	*P* value
Lg[sLYVE-1] per SD	1.733 (1.321–2.272)	0.000	1.733 (1.321–2.272)	0.000	1.633 (1.144–2.330)	0.007
sLYVE-1 per SD	1.804 (1.361–2.392)	0.000	1.804 (1.361–2.392)	0.000	1.706 (1.167–2.493)	0.006

Patients were classified as eGFR < 60 mL/min/1.73 m^2^ group and eGFR > 60 mL/min/1.73 m^2^ group. sLYVE-1 was analyzed as a log transformed continuous variable. The unit of change was specified as per SD for Lg (sLYVE-1) (per SD = 0.215) and for sLYVE-1(per SD = 261.00). Model 1: adjusted for gender, age. Model 2: adjusted for gender, age, BMI, SBP, Fasting glucose, HbA1c, LVEF, hsCRP, HBP and DM. BMI, body mass index; SBP, systolic blood pressure; LVEF, left ventricular ejection fraction; hsCRP, high sensitivity C reactive protein; HBP, high blood pressure; DM, diabetes mellitus.

## Discussion

### The role of LYVE-1 in pathological conditions has not been fully identified

LYVE-1, which has 41% sequence homology to the HA receptor CD44, has been proved to play an important role in HA clearance and HA-mediated leukocyte adhesion^7^. However, a number of studies have showed that LYVE-1 is also expressed in certain bone marrow-derived monocytic lineages, which probably function in lymphangiogenesis in pathological processes including chronic inflammatory diseases, transplant rejection, and tumorigenesis^25–28^.

The results of studies in which the LYVE-1 gene was deleted in mice have been controversial. Nicholas *et al*.^29^ and Mai *et al*.^30^ showed that LYVE-1 knock out (LYVE-1^−/−^) mice exhibited an clearly normal phenotype, without any detected significant changes in lymphatic vessel ultrastructure or function. In addition, LYVE-1^−/−^ mice also showed typical trafficking of dermal CD11c^+^ dendritic cells to draining lymph nodes via afferent lymphatics and normal regression of drug-induced skin inflammation. Conversely, Johnson *et al*.^31^ reported that LYVE1 gene deletion not only impeded lymphatic delivery of cutaneous dendritic cells but also impaired their ability to trigger CD8^+^ T cell responses in skin-draining lymph nodes. These results suggest that LYVE-1 might not be essential for normal lymphatic development and function, but do not rule out a role for LYVE-1 in pathological conditions such as chronic inflammation.

### LYVE-1 ectodomain shedding

The ectodomain of LYVE-1 is known to undergo proteolytic cleavage, producing the soluble form of LYVE-1. This process can be provoked by vascular endothelial growth factor (VEGF-A), which is an important growth factor promoting lymphangiogenesis and angiogenesis in pathological conditions. Surprisingly, wild-type LYVE-1, but not uncleavable LYVE-1(generated by a mutation at the site of proteolytic cleavage), facilitated migration of lymphatic endothelial cells in response to VEGF-A, indicating a potential role of ectodomain shedding of LYVE-1 in accelerating pathological lymphatic vessel growth^10^. Furthermore, Hoi Leong Xavier *et al*. reported that LYVE-1-mediated lymphangiogenic responses can be inhibited by membrane type 1-matrix metalloproteinase (MT1-MMP), an endogenous suppressor of lymphangiogenesis, which directly cleaves LYVE-1 on lymphatic endothelial cells^32^. Consistently, it has been revealed that sLYVE-1 impairs fibroblast growth factor 2 (FGF2)/LYVE-1 signaling and functions^11^. These data suggest an antagonistic effect of sLYVE-1 on LYVE-1 signaling in lymphatic endothelial cells.

### The value of sLYVE-1 in diagnostics

The association of sLYVE-1 with several pathological conditions has been reported, and an increase in sLYVE-1 levels during inflammation and tumorigenesis has been observed^12,13^. However, in the current study, serum sLYVE-1 concentrations showed no significant differences between subjects with chronic pathological conditions including hypertension and dyslipidemia and those without. Unexpectedly, a patient’s sLYVE-1 level was not associated with the occurrence and severity of CAD. Although the interpretation of the negative results should be carefully made because of small test powers, these data suggest that changes in sLYVE-1 level do not occur in all pathological conditions. Significant changes might only occur when the balance of activators and inhibitors of LYVE-1 ectodomain shedding is completely disturbed.

### The role of lymph system in CAD remains an interesting area worth exploring

Although sLYVE-1 levels were not associated with the occurrence and severity of CAD, the role of the lymphatic system in CAD remains an interesting area. Recently, lymphatic vessels have been reported to play an important role in reverse cholesterol transport and in the regression of atherosclerosis in experimental animal models. Mouse strains with lymphatic insufficiency have higher plasma cholesterol levels in comparison with control mice fed both a western-type high fat diet and a normal diet^15^. Thus, therapies aimed at reversing atherosclerosis may benefit from cholesterol clearance mediated by lymphatic transport function^14^.

It has been reported that ApoE^−/−^ mice display an increase in lymphangiogenesis in progressive atherosclerotic lesions characterized by calcium deposits and cholesterol crystals, and in atherosclerotic iliac arteries^5,33^. A change of adventitial lymphatics in the progression of atherosclerosis has also been reported^34,35^. It is intuitive that deficient lymphatic drainage could aggravate atherosclerosis by impeding the clearance of excessive amounts of lipids, inflammatory cytokines, and immune cells. Thus, an abnormal angiogenesis and lymphangiogenesis might be a part involved in the sustained inflammatory response during human atherogenesis.

### The effect of sLYVE-1 on renal dysfunction remains unidentified

In the present study, a significant association of sLYVE-1 and renal dysfunction was, for the first time, observed. An increased sLYVE-1 level was an independent risk factor for lower eGFR. These data imply changes in the lymphatic system in patients with renal dysfunction.

The expression pattern of LYVE-1 in mouse kidney has been illustrated by Hyun-Wook and his colleagues^36^. In the adult mouse kidney, LYVE-1 was predominantly located in the lymphatic endothelial cells and in certain endothelial cells in the glomerular capillaries. Under pathological conditions in a mouse model of renal interstitial fibrosis, an increase of lymphangiogenesis in the fibrotic kidney has been observed^37,38^. Meng *et al*.^39^ reported that after renal ischemia and reperfusion, LYVE-1 was localized in the endothelial cells of the glomerular region. Taking all these together, the roles of LYVE-1 and sLYVE-1 appear to important and are not yet completely understood.

In conclusion, our data show the serum sLYVE-1 level in different pathological conditions, and reveal a positive association with renal dysfunction. Although the exact role of sLYVE-1 in disease progression needs to be further elucidated, renal function should considered when sLYVE-1 is used as a biomarker when diagnosing pathological conditions in the future.
